# Cytokine storm during chemotherapy: a new companion diagnostic emerges?

**DOI:** 10.18632/oncotarget.27442

**Published:** 2020-01-21

**Authors:** Panagiota S. Filippou, George S. Karagiannis

**Keywords:** cytokines, chemotherapy, diagnostics

Despite that chemotherapy represents the frontier cancer treatment today, aggregating evidence in pre-clinical animal models and small patient cohorts paradoxically suggests that certain cancers often elicit pro-tumorigenic and pro-metastatic phenotypes in response to standard chemotherapy regimens. This phenotype is now believed to occur through a cancer-mediated secretion of pro-inflammatory cytokines, chemokines and other mediators, collectively known as the “cytokine storm”, initially affecting the local tumor microenvironment, but subsequently released in peripheral circulation, where it elicits systemic tumor-promoting effects [1]. Two recent, seminal studies in this context, the first by Karagiannis et al. (2017) in Science Translational Medicine [2], and the second by Gartung et al. (2019) in Proceedings of the National Academy of Sciences (PNAS) [3], have shed useful insights on the molecular mechanisms underlying the pro-tumoral shift of the tumor microenvironment upon treatment with chemotherapy. When appreciated individually, each study reveals a unique piece of the puzzle, but valued together, they offer an attractive model on how “chemotherapy-induced metastasis” is regulated at the microanatomical level.

Generally, tumor-associated macrophages (TAMs) represent a proponent compartment of the tumor microenvironment and they are well-documented to promote tumor angiogenesis, metastasis, and local immunosuppression, as well as to obscure tumor response to various cancer treatment modalities, including chemotherapy, immunotherapy and targeted antiangiogenic therapy [1]. Of note, a specialized TAM subpopulation exerting M2 (or M2-like) polarization status that expresses the tyrosine kinase receptor Tie2 (Tie2^+^ TAMs) has been recently identified as the master regulator of tumor angiogenesis and cancer cell intravasation and dissemination. Interestingly, Tie2^+^ macrophages do not only regulate cancer angiogenesis, but they have been notorious for assembling tripartite micro-anatomical doorways called Tumor Microenvironment of Metastasis (TMEM), which are composed of one tumor cell overexpressing the actin-regulatory protein Mammalian enabled (Mena), one pro-angiogenic Tie2^+^ macrophage, and one underlying endothelial cell, all three in direct physical juxtaposition to one another. Such TMEM doorways function as active sites of cancer cell dissemination, and they can literally gunfire highly-migratory, highly-invasive tumor cells into the peripheral circulation. TMEM activity is conveniently regulated by the Tie2^+^ TAM through the local secretion of vascular endothelial growth factor-A (VEGF-A), and as such, tumors with high TMEM density and activity have an increased risk for developing metastatic disease [1]. In the seminal study by Karagiannis et al. (2017), it was shown that preoperative (i.e., neo-adjuvant) chemotherapy with taxane (i.e., paclitaxel) or non-taxane (i.e., doxorubicin/cyclophosphamide) chemotherapeutics induced the recruitment of Tie2^+^ macrophages in the primary breast tumor microenvironment, subsequently promoting TMEM doorway assembly and function, leading to a prominent increase of circulating tumor cells and metastatic dissemination and disease [2]. Similar (or relevant) observations have been described by other research groups ever since [for detailed description on those studies refer to Sanchez et al. (2019) [1], and the references within], collectively suggesting that chemotherapy-driven metastasis is mediated by influx of pro-metastatic myeloid cells (specifically macrophages) and endothelial progenitors.

Nevertheless, an unresolved, yet critical, question rising from Karagiannis et al. (2017) at the time was related to the precise mechanism via which Tie2^+^ macrophages were seen to be recruited in primary breast tumors upon treatment with neoadjuvant chemotherapy. Prior literature [1], along with the key observation that identical TMEM-mediated pro-metastatic responses were not dependent on the category of the chemotherapeutic used [2], prompted the idea that Tie2^+^ TAM influx is the cumulative result of a host repair-mechanism against cancer cell apoptosis, hypoxia and extensive tissue damage, as elicited by the cytotoxic nature of chemotherapy. This speculation has been confirmed in the elegant study by Gartung et al. (2019), which profiled the pro-inflammatory mediators released upon treatment with neo-adjuvant chemotherapy in ovarian cancer [3]. Strikingly, ovarian cancer cell debris resulting from cytotoxic tissue damage was capable *per se* to elicit therapy-induced tumor progression, by enforcing the release of the cytokine storm systemically [3]. This study highlighted that circulating levels of tumor necrosis factor-alpha (TNF-α), C-C motif chemokine ligand-2 (CCL2), and interleukin 6 (IL-6) were significantly increased upon chemotherapy, and suggested that these cytokines could drive chemotaxis of other cytokine-releasing stromal/immune cells, thus perpetuating a pro-inflammatory vicious cycle supporting hallmarks of tumor growth and progression [3]. Certain cytokines, such as the ones mentioned above, have been demonstrated to be involved in monocyte/macrophage chemotaxis, infiltration, maturation, and their functional implications within the tumor microenvironment [1], and more importantly, Gartung et al. (2019) identified and validated these key cytokines using unbiased high-throughput analyses [3].

In general, cytokines are molecules with key roles in intercellular communication within the tumor microenvironment, and certain of them have already been proposed as therapeutic cancer targets [4]. Interestingly, cancer cells and the tumor microenvironment can create a surge in cytokine production, termed “cytokine storm”, upon treatment with chemotherapy [1, 3, 4], as described above. These cytokines are not only responsible for generating a tumor- and metastasis-promoting microenvironment in cancer, including the establishment of the pre-metastatic niche, but may also be involved in drug resistance and chemoresistance. Cytokines modulate the release of growth factors, or even other cytokines, thus perpetuating a pro-inflammatory, pro-invasive and pro-metastatic loop within the tumor microenvironment. Cancer cells readily respond to such host-derived cytokines to acquire designated hallmarks of cancer, including survival, apoptosis evasion, invasion and metastasis [4]. Because of such debilitating and promiscuous functions, cytokine identification and quantification in both the local or distant tumor microenvironment and in the blood circulation have been at the frontier of translational research, which examines their potential application as diagnostic, prognostic, or predictive biomarkers. Indeed, technological advancements and innovative, state-of-the art analytical approaches have granted us the ability to study and measure this complex cytokine network at the systemic level. A wide range of cytokine assays is available nowadays: i) direct measurement of cytokines or indirect measurement of cytokine soluble receptors in body fluids or cellular supernatants (immunoassays, cytokine bioassays, western blot, mass spectrometry); ii) cytokine measurement produced by cell populations (multiparametric flow cytometry, magnetic bead-based quantitation of cytokine producing cells, mRNA-based assays); iii) single cytokine-producing cell analysis (ELISpot, flow cytometry, mass cytometry, emerging techniques for single cell secretomics and droplet-based microfluidic approaches); and finally, iv) cytokine imaging at the cellular and tissue levels (immunohistochemistry, immunofluorescence, intra-cytoplasmic cytokine staining (ICC)) [5].

Given the abundance of the techniques described above, the measurement of individual cytokines should, in theory, be easily measured in tissues and biological fluids. Despite the aforementioned advances however, the accurate and reproducible cytokine measurement still faces considerable challenges, which typically include their context-dependent expression and release, their often low concentrations (picomolar levels or lower) in biological fluids, and their binding and/or interference with other proteins in many common analytical assays [5]. One would therefore advocate for the development of novel, or the improvement of current, analytical techniques that simultaneously measure multiple cytokines (e.g., components of the cytokine storm) in the form of “cytokine panels”, which are more informative and reflective of the true biology and pathology of the disease. With the ever-increasing understanding of the complex circuitries and interactions within the tumor microenvironment, such multiplexing appears to be an analytical strategy that will dominate in the future. A new era in proteomics and other bioanalytical techniques brings this new vision forward with innovative and/or ultrasensitive techniques [i.e., multiplex immunoassay platforms; mass-spectrometry (MRM-MS); chemi-luminescent, bead-based (Luminex) and planar antibody arrays; Singulex; Simoa; immuno-PCR; proximity ligation/extension assay and immunomagnetic reduction assay] [5].

Knowing the devastating effects of the cytokine storm released upon chemotherapy, as previously described [1–3] such powerful tools could be developed in the future to assist clinicians in monitoring prometastatic responses in patients in the course of treatment with neo-adjuvant chemotherapy. Indeed, Karagiannis et al. (2017) suggested that breast cancer patients receiving neo-adjuvant chemotherapy and not achieving pathologic, complete or partial, response (pCR) attract significantly more Tie2^+^ macrophages assembling TMEM doorways, thus putting this patient subpopulation in a high-risk group of developing distant metastasis later in their lifetime [2]. In a complementary fashion, Gartung et al. (2019) has identified and quantified certain components of the chemotherapy-elicited cytokine storm that function as perpetrators of this phenotype [3]. Unfortunately, these studies [2, 3] also underscore our inability, as of the moment, to predict which patients will respond with pCR upon treatment with neo-adjuvant chemotherapy, thus making the analytical strategies described here as perhaps the most promising tools for guiding future clinical decisions in patient management and care. We therefore anticipate that multiplex cytokine signatures will be used as companion diagnostics for current therapeutic modalities, thus allowing for more “precision” in the era of personalized medicine.
